# Compulsive Skin Picking in Anorexia Nervosa: A Qualitative Study of Emotional and Interoceptive Dysregulation

**DOI:** 10.3390/nu18071070

**Published:** 2026-03-27

**Authors:** Jaworski Mariusz, Giersz Urszula

**Affiliations:** 1 Department of Education and Research in Health Sciences, Faculty of Health Science, Medical University of Warsaw, 00-581 Warsaw, Poland; 2 Faculty of Health Sciences, Medical University of Warsaw, 00-581 Warsaw, Poland; ula.giersz@wp.pl

**Keywords:** anorexia nervosa, body-focused repetitive behaviors, skin picking, interoception, energy restriction, emotional regulation, eating disorders, hunger-related stress, multidisciplinary treatment

## Abstract

Background: Body-focused repetitive behaviors (BFRB), including compulsive skin picking, are observed in patients with anorexia nervosa (AN). However, their functional significance remains unclear. AN is characterized by chronic energy restriction and altered interoceptive processing, which may contribute to persistent internal tension beyond overt eating symptoms. This study aimed to explore the functional meaning of compulsive skin picking as a potential behavioral marker of emotional and interoceptive dysregulation relevant to clinical assessment and treatment planning. Methods: A qualitative descriptive study was conducted among 33 hospitalized patients with AN who reported recurrent skin picking leading to tissue damage. Patients were undergoing structured nutritional rehabilitation. Individual semi-structured interviews were performed. Results: Patients with AN described a consistent emotional and physiological sequence preceding skin picking: increased internal tension followed by short-term relief and subsequent self-directed negative emotions. Episodes occurred in contexts of emotional overload, understimulation, reduced emotional awareness, and, in some cases, hunger- or meal-related stress. Participants described the behavior as partly intentional, serving to reduce tension, but at times occurring almost automatically. It was closely connected with eating-related anxiety and dissatisfaction with the body. Conclusions: The findings indicate that compulsive skin picking in AN may reflect underlying emotional and interoceptive instability, instead of being an unrelated co-occurring symptom. In the broader context of chronic energy restriction, such behaviors may reflect attempts to modulate internally generated physiological arousal. Incorporating routine assessment of BFRB into clinical practice could improve the identification of ongoing emotional instability and support more coordinated multidisciplinary care. Future studies combining qualitative insights with physiological measures would help clarify the mechanisms underlying this association.

## 1. Introduction

Anorexia nervosa (AN) is a severe psychiatric disorder characterized by disturbed body image and restrictive eating patterns. It remains one of the psychiatric conditions with the highest mortality rates and represents a substantial burden for healthcare systems worldwide [1]. In addition to its psychological features, AN is associated with chronic energy deficiency and persistent alterations in physiological homeostasis, including endocrine and metabolic adaptations [2]. Although clinical improvement is frequently evaluated through weight restoration and somatic stabilization [1], many patients continue to experience disturbances in emotional processing and internal state regulation even when nutritional parameters begin to normalize [3,4].

Beyond its psychological manifestations, AN is fundamentally associated with prolonged caloric restriction and sustained biological signals of hunger. Chronic energy deficiency activates neuroendocrine stress pathways, including the hypothalamic–pituitary–adrenal axis and autonomic adaptations [2], and has been associated with altered interoceptive processing in individuals with AN [5,6]. Hunger-related physiological arousal may be experienced as irritability, inner tension, and restlessness, consistent with evidence that acute hunger increases negative affective states [7]. In individuals with AN, these biologically driven signals may conflict with rigid cognitive control over eating behavior, consistent with evidence that cognitive overcontrol is a characteristic feature of the disorder [8]. This psychobiological conflict between homeostatic needs and overcontrolled behavior may contribute to persistent internal tension that cannot be resolved through adaptive nutritional responses.

In this context, individuals with AN may experience pronounced difficulties in recognizing and regulating internal states, particularly when bodily signals conflict with cognitive control over eating. Such conditions may increase reliance on maladaptive behavioral strategies aimed at reducing internal tension [3,5,6,9].

In multidisciplinary treatment settings, identifying behavioral signs of ongoing dysregulation may be especially valuable [3]. Treatment decisions made by physicians, psychotherapists, nurses, and dietitians frequently depend on self-reported experiences. However, self-report may be limited by reduced emotional awareness, alexithymia, or avoidance of internal states [10]. Observable behaviors that reflect attempts at regulation may therefore add clinically relevant information and support more precise case formulation.

Body-focused repetitive behaviors (BFRB), including compulsive skin picking (excoriation disorder) [11,12], are characterized by repetitive, difficult-to-control actions directed toward one’s own body, typically emerging in states of heightened internal tension and followed by short-term relief, consistent with a negative reinforcement pattern [13,14].

From a nutritional perspective, skin integrity is strongly influenced by the availability of essential nutrients, including fatty acids, vitamins (e.g., vitamin A), and micronutrients involved in tissue repair and immune function. Deficiencies in these components, which are common in individuals with anorexia nervosa due to prolonged dietary restriction, may contribute to impaired skin barrier function and increased vulnerability to skin damage. This aspect is particularly relevant in the context of anorexia nervosa, where prolonged malnutrition may affect both skin physiology and overall somatic functioning [15,16].

Although BFRB have been studied primarily in terms of prevalence and diagnostic classification, their role within eating disorder psychopathology remains insufficiently explored. Emerging evidence suggests that BFRB may co-occur with eating disorders and share common underlying mechanisms, particularly difficulties in emotion regulation, body-focused attention, and maladaptive coping strategies [11,12,17]. This overlap is particularly relevant given that both conditions involve heightened body-focused attention and difficulties in emotion regulation. Recent studies also suggest that body-focused repetitive behaviors may be associated with eating disorder symptomatology, particularly in relation to emotion regulation difficulties and body image disturbances [12,13,14].

However, relatively little is known about the functional and phenomenological meaning of compulsive skin picking specifically in individuals with anorexia nervosa, particularly in the context of interoceptive dysregulation and chronic energy restriction.

Chronic autonomic arousal associated with energy deficit and altered interoceptive processing in AN may increase vulnerability to alternative tension-regulation behaviors [2,5,6].

When eating, one of the primary biological mechanisms restoring homeostasis is cognitively restricted, and internal tension may seek alternative behavioral outlets. Within this perspective, compulsive skin picking can be understood as a small-scale regulatory attempt to reduce physiological and affective arousal that remains unresolved. This interpretation aligns with negative reinforcement mechanisms described in the BFRB literature [13,14].

While interoceptive dysfunction in AN has been increasingly examined [5,6], relatively little attention has been paid to whether BFRB might serve as secondary responses to biologically driven tension associated with starvation and body-related anxiety. Comorbidity between eating disorders and body-focused repetitive behaviors has been reported in both clinical and epidemiological studies [11,12]. However, most investigations have concentrated on prevalence rates and diagnostic categorization, with less emphasis on understanding the functional role of these behaviors within the metabolic and interoceptive context of anorexia nervosa.

Disturbances in body image represent a core feature of AN and shape both emotional processing and coping patterns [3,4]. Heightened self-focused appearance monitoring and persistent body dissatisfaction may further amplify attention to perceived skin imperfections. From this standpoint, compulsive skin picking may be embedded in distorted body image processing rather than occurring as an independent comorbid condition. The behavior may simultaneously reflect affect regulation and attempts to modify perceived bodily flaws, linking interoceptive arousal, emotional tension, and appearance-focused control mechanisms.

Addressing this gap may have practical implications. If compulsive skin picking represents an observable marker of ongoing emotional and interoceptive dysregulation, its routine assessment may improve clinical evaluation, particularly in contexts where reduced emotional awareness and alexithymia limit the reliability of self-report [10]. Understanding the functional role of BFRB in AN may also inform interventions targeting emotion regulation and interoceptive processing [3,9].

The aim of the present study was therefore to explore the functional meaning of compulsive skin picking in individuals with anorexia nervosa within a clinical context. The study sought to explore whether compulsive skin picking may serve as an observable marker of emotional and interoceptive dysregulation and to analyze its relevance for clinical assessment and multidisciplinary treatment planning. Particular attention was given to cognitive–emotional mechanisms accompanying the behavior and its relationship to eating disorder symptomatology and treatment experience.

The analysis focused on several key experiential domains:

1. Emotional and interoceptive states occurring before and after the behavior, including physiological arousal and hunger-related sensations;
2. Cognitive interpretations and meanings attributed to the behavior;
3. Situational and contextual triggers, including eating-related and treatment-related circumstances;
4. The perceived regulatory function of the behavior (emotional, sensory, and tension-modulating aspects);
5. The perceived relationship between the behavior and eating disorder symptom severity, nutritional status, and treatment experience.

## 2. Materials and Methods

### 2.1. Study Design and Methodological Approach

The study employed a qualitative descriptive design [18] with thematic analysis [19]. Individual semi-structured interviews were conducted to enable an in-depth exploration of the meanings attributed to compulsive skin picking behavior by individuals diagnosed with AN. This methodological approach was selected due to the exploratory nature of the research problem.

### 2.2. Approach Participants and Sampling Criteria

The study included patients with AN hospitalized in the Department of Pediatrics, Nutrition, and Metabolic Diseases at a clinical hospital in Warsaw. Patients were enrolled in a structured nutritional rehabilitation program that included supervised meals and ongoing medical monitoring of somatic status. At the time of the interviews, patients were at different stages of weight restoration.

Purposive sampling [20] was applied. Inclusion criteria were as follows: (1) a current diagnosis of AN; (2) the presence of recurrent skin picking behaviors resulting in tissue damage; (3) age ≥ 16 years; and (4) the capacity to provide informed consent and participate consciously in the study.

Exclusion criteria included: (1) a somatic condition precluding safe participation in the interview (e.g., severe malnutrition, electrolyte disturbances, syncope, or the need for intensive medical care); (2) a psychiatric condition that could impair the ability to participate in the interview (including severe depressive symptoms with suicidal risk, psychotic symptoms, disturbances of consciousness, or marked agitation); (3) inability to provide informed consent; (4) failure to meet inclusion criteria, including absence of a diagnosis of anorexia nervosa or absence of dermatillomania-related behaviors; and (5) other clinical or organizational barriers preventing the conduct of the interview (e.g., premature discharge or inability to ensure confidentiality).

Recruitment continued until thematic saturation was achieved [21], defined as the point at which no new substantive categories emerged in three consecutive interviews. Saturation was assessed independently by both coders and confirmed through consensus discussion.

### 2.3. Research Instrument: Semi-Structured Clinical Interview

Data were collected using a semi-structured clinical interview developed specifically for the purposes of this study. The interview guide comprised 12 open-ended questions organized into the following thematic domains: (1) the course of the eating disorder and previous treatment experiences; (2) sociodemographic characteristics; (3) characteristics of compulsive skin picking behaviors; (4) emotional, cognitive, and interoceptive experiences, including bodily sensations related to hunger, arousal, and somatic discomfort, as well as situational contexts accompanying the behavior; and (5) the perceived regulatory function of the behavior.

The interview structure ensured comparability of the collected data while preserving space for participants’ spontaneous narratives. Questions were exploratory in nature and focused on describing the course and function of the behavior rather than on its formal diagnostic assessment.

### 2.4. Study Procedure

Patients received both oral and written information regarding the study objectives, the voluntary nature of participation, and the right to withdraw at any stage without consequences. Interviews were conducted individually in a confidential setting within a therapeutic room of the Department of Pediatrics, Nutrition, and Metabolic Diseases. The interviewer was not directly involved in the therapeutic decision-making process concerning the participants.

Interviews lasted between 25 and 45 min (mean duration: 32 min). They were conducted by a researcher trained by a clinical psychologist and psychotherapist in conducting clinical interviews and responding to patients’ emotional discomfort. The training included principles of clinical interviewing, recognition of psychological distress signals, techniques of emotional support, and ethical standards applicable to clinical practice. The researcher was prepared to conduct interviews in an empathetic, non-judgmental manner, with full respect for participants’ boundaries and psychological well-being. Additionally, she was trained in managing situations involving heightened emotional tension and in procedures for safely interrupting the interview if required by the participant’s condition. Ensuring participants’ psychological safety was prioritized throughout the interview process.

The entire procedure was supervised by a clinical psychologist responsible for substantive oversight of the project, thereby ensuring full safety and comfort of participants in accordance with ethical standards for research involving hospitalized patients.

Given the potential for increased tension among patients with anorexia nervosa, interviews were not audio-recorded. Instead, detailed field notes were taken contemporaneously during each interview and expanded immediately afterward. These notes included quasi-verbatim excerpts of participants’ statements, contextual descriptions, observations of emotional tone, and relevant nonverbal behaviors.

To enhance credibility, each interview summary was reviewed shortly after data collection within the research team, and ambiguities were clarified while the interaction remained recent in memory. A structured documentation procedure was followed across all interviews to ensure procedural consistency. This approach is consistent with qualitative research recommendations in psychiatric settings [22] and is considered acceptable when audio recording may influence participant behavior. To reduce bias, the material was analyzed independently by two researchers.

### 2.5. Data Analysis

Thematic content analysis was conducted in accordance with the procedure described by Braun and Clarke [19]. As interviews were not audio-recorded, due to considerations related to participants’ psychological comfort and the specific clinical setting, where recording could have induced anxiety or behavioral inhibition, detailed field notes constituted the primary source of data. These notes included verbatim excerpts of statements as well as descriptions of contextual factors, emotional reactions, and nonverbal cues.

The analysis proceeded through six stages: (1) Familiarization with the data—Repeated reading of all field notes, and contextual observations. (2) Initial coding (open coding)—Identification of recurring themes, emotions, thoughts, situations, and responses. (3) Categorization—Grouping similar codes into preliminary thematic clusters. (4) Theme development—Organizing categories into broader analytical domains (e.g., “emotions preceding the behavior,” “regulation of tension,” “somatic symptoms”). (5) Review of coherence—Comparison of themes with the source material to ensure consistency and to avoid overinterpretation. (6) Reporting–Description of categories and themes supported by representative quotations.

An initial coding framework was developed independently by two researchers experienced in qualitative analysis and clinical work in eating disorders. Following independent coding of the initial set of interviews, codes were compared and discussed to establish a shared analytic structure. Discrepancies were resolved through discussion until consensus was reached. A coding log was maintained throughout the analytic process to document analytic decisions, theme development, and category refinement. This audit trail enhanced transparency and minimized the risk of overinterpretation.

Interviews were conducted by a researcher specifically trained for this project by an experienced clinical psychologist and psychotherapist. The training covered standards of clinical interviewing, recognition of distress signals, safeguarding psychological well-being, and ethical principles of qualitative research. The clinical psychologist supervised the entire procedure to ensure adherence to ethical and clinical standards applicable to hospitalized patients with anorexia nervosa.

### 2.6. Ensuring Quality and Trustworthiness

Credibility of the analysis was ensured through independent coding by two researchers and resolution of discrepancies through consensus. A standardized interview protocol and supervision by a clinical psychologist were implemented to enhance methodological rigor. A detailed description of the clinical context and study population was provided to facilitate assessment of transferability.

Qualitative research criteria [23] were applied, including independent coding, reflexive discussions, maintenance of an audit trail, and comprehensive contextual description supporting transferability of findings.

The research team acknowledged the potential for interpretative bias associated with their clinical background and prior experience in the treatment of eating disorders. Reflexive discussions were conducted throughout the analytic process to minimize overinterpretation and maintain descriptive fidelity.

### 2.7. Ethical Considerations

The study was conducted in accordance with the Declaration of Helsinki and approved by the Bioethics Committee of the Medical University of Warsaw (approval no. AKBE/393/2025, 8 December 2025). All field notes were anonymized immediately after each interview and stored in password-protected electronic files accessible exclusively to the research team. For participants under 18 years of age, written informed consent was obtained from their legal guardians in addition to the participant’s assent.

## 3. Results

### 3.1. Characteristics of Participants

A total of 39 individuals with a confirmed history of anorexia nervosa were enrolled in the study. The majority of participants were female (94.9%) and ranged in age from 16 to 44 years. However, for the purposes of qualitative analysis, a subgroup of patients presenting body-focused repetitive behaviors (BFRB), including compulsive skin picking, was identified (n = 33). Of the 39 interviewed patients, 33 reported recurrent skin picking episodes that met the predefined criteria for BFRB and were therefore included in the thematic analysis. The remaining six participants did not report recurrent skin picking behaviors fulfilling the predefined BFRB criteria and were consequently excluded from further thematic analysis.

The analyzed subgroup (n = 33) consisted exclusively of female patients with AN (100%). Participants ranged in age from 16 to 44 years, with the largest proportion aged 18–24 years (54.5%), followed by 25–34 years (30.3%) and 16–18 years (15.2%).

Regarding clinical characteristics, 26 patients (78.8%) were diagnosed with anorexia nervosa without comorbid eating disorders, whereas 7 (21.2%) presented comorbid eating disorders (bulimia nervosa, n = 4; binge-eating episodes, n = 2; orthorexia, n = 1).

In terms of educational level, 57.5% had higher education, 27.3% secondary education, and 15.2% primary education. Most patients resided in cities with more than 200,000 inhabitants (57.5%), while 18.2% lived in cities with 50,000–200,000 inhabitants, 9.1% in cities with fewer than 50,000 inhabitants, and 15.2% in rural areas. Detailed sociodemographic and clinical characteristics are presented in Table 1. All participants were diagnosed with anorexia nervosa by a psychiatrist according to ICD-10 criteria within a clinical setting. Detailed anthropometric parameters (e.g., body weight, height, BMI) were not systematically collected for the purposes of this qualitative study, as the primary focus was on experiential and behavioral aspects rather than somatic or nutritional profiling.

### 3.2. Core Emotional Regulation Mechanism of Compulsive Skin Picking

Numerical frequencies are provided to illustrate the relative prominence of thematic categories. However, the analysis remains qualitative and interpretative in nature. All participants (n = 33) reported experiencing distinct emotional states both prior to and following episodes of skin picking. Narrative analysis revealed recurrent emotional transitions between antecedent states and the immediate consequences of the behavior. These transitions were characterized by fluctuations in emotional and physiological arousal, followed by the emergence of secondary negative emotions.

Several participants described this process as a recurring internal cycle of tension and relief:

*“I feel like the tension builds up and I cannot ignore it. When I start picking, it gives me a sense of relief, but it only lasts for a short time. Afterwards, I feel worse because I know I have done it again.”*

Another participant described a similar pattern:

*“For me, it’s like something accumulates inside and I can’t release it in any other way. I try to ignore it, but the tension keeps growing. When I start picking, it gives me immediate relief, but then I feel guilty and frustrated because I know it doesn’t really solve anything. It becomes a kind of loop, the tension builds up again, and I feel the urge to repeat it. In that moment, it feels like the only way to reduce what I’m experiencing, even though I know it’s not helpful in the long term.”*

The most consistent pattern involved heightened emotional and physiological arousal preceding the behavior, followed by a short-term reduction in internal tension and the subsequent appearance of secondary negative affective states.

Each identified pattern was observed across multiple participants rather than derived from isolated statements. In total, seven dominant patterns of emotional transitions were identified. The patterns presented in Table 2 illustrate recurring emotional sequences described by participants. The accompanying quotations reflect typical narrative accounts rather than singular or exceptional statements.

**Figure 1:**
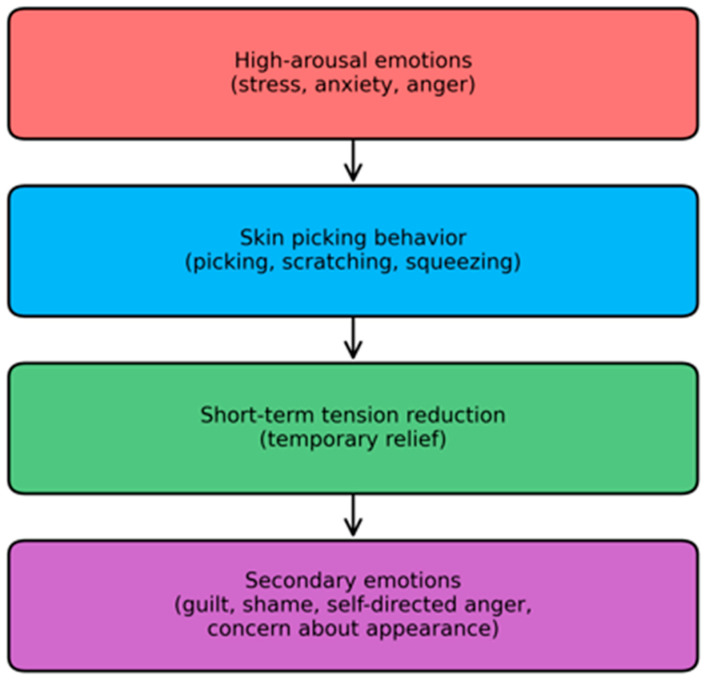
Schematic representation of the emotional sequence reported by participants.

Episodes of skin picking were typically preceded by heightened emotional arousal, followed by a transient reduction in internal tension and the subsequent emergence of self-directed negative emotions (e.g., guilt, shame, and concerns related to physical appearance) (see Figure 1).

### 3.3. Antecedents of the Behavior

The antecedents of skin picking clustered into three primary domains: (1) emotional triggers, (2) situational context, and (3) physiological arousal.

#### 3.3.1. Emotional Triggers

All patients described some form of internal state preceding episodes of skin picking. Although the emotional tone differed across AN patients, most accounts pointed to a similar experience: a sense of discomfort or difficulty remaining with the current internal state.

The most commonly described antecedents involved heightened arousal, including tension, anxiety, frustration, or anger. Patients with AN referred both to acute stress and to more prolonged emotional strain.

*“Before—stress and agitation.”*

*“Anxiety, irritation, inner pressure.”*

*“Anger when I see imperfections.”*

These experiences were sometimes described in more detail as follows:

*“I feel tension building up inside me, like I cannot stay with it. It can be stress, anxiety, or irritation, and I start focusing on my skin. When I begin picking, it helps me release that tension for a moment, but afterwards I feel frustrated that I did it again.”*

In several cases, the perceived imperfections concerned specific body areas. For these AN patients, skin picking appeared to be closely tied to appearance-related concerns rather than to diffuse emotional tension alone.

Another group of antecedents emerged in contexts of reduced stimulation or low arousal, such as boredom, fatigue, or a need to occupy attention.

*“Before—boredom and emptiness.”*

*“I did it when nothing was happening.”*

In some cases, patients with AN reported no identifiable emotion preceding the behavior and described the episode as occurring automatically.

*“Nothing specific… it just happened.”*

*“I started without noticing.”*

Across patients, the behavior was consistently preceded either by heightened arousal, understimulation, or an unrecognized internal state. These antecedent conditions were consistently reported across participants and did not represent isolated accounts. The identified antecedents correspond to the emotional transitions described in Section 3.2.

The reported emotional antecedents are summarized in Table 3. The table presents recurring categories of internal states preceding skin picking episodes along with representative participant quotations.

In several narratives, patients with AN spontaneously linked episodes to hunger, food-related anxiety, or meal-related stress.

#### 3.3.2. Situational Context

Patients described recurring situational contexts in which episodes of skin picking occurred (see Table 4). The behavior most often appeared during emotionally demanding circumstances, including interpersonal stress, mental overload, or fatigue. Episodes were also commonly reported during periods of reduced activity, particularly in the evening, before sleep, or while engaging in passive activities requiring little attention.

Some patients indicated that the behavior occurred when their hands were unoccupied or during monotonous tasks. The majority of episodes took place in private settings, most commonly at home or in a personal room. In several cases, skin picking was associated with rumination or a subjective sense of loss of control, whereas some participants reported no specific situational pattern. These contexts were reported by multiple participants and did not represent isolated situations.

#### 3.3.3. Physiological and Interoceptive Arousal

Patients with AN reported physiological sensations accompanying or directly preceding skin picking episodes. A substantial proportion did not identify clear bodily sensations and described the behavior as occurring without conscious awareness. Among the remaining patients, symptoms of autonomic arousal were reported, including increased heart rate, muscle tension, and sensations of heat or sweating. Less frequently, participants described more complex activation symptoms such as dizziness or breathing discomfort. The reported bodily sensations are summarized in Table 5. Some participants described bodily sensations consistent with heightened autonomic activation, while others reported reduced awareness of internal states.

### 3.4. Immediate Consequences

Patients with AN were asked whether skin picking influenced their emotional state or coping with tension. Most patients with AN reported a temporary change in emotional state following the behavior, although the perceived effects varied. Some individuals described a short-term reduction in tension, while others reported no change or ambivalent effects. A small group indicated that the behavior intensified emotional discomfort. Perceived effects of the behavior are presented in Table 6.

Patients with AN also described specific emotional experiences occurring after the episode. These experiences are summarized in Table 7.

### 3.5. Cognitive Experience During Behavior

Patients described thoughts occurring during episodes of skin picking. The reported cognitive experiences varied from the absence of conscious thoughts to repetitive or intrusive cognitions focused on the body, tension reduction, or the ongoing act. Some patients reported automatic engagement without identifiable thoughts, whereas others described urge-related cognitions, self-evaluative thoughts, or attempts to control internal states. The identified categories of cognitive experiences are presented in Table 8.

### 3.6. Behavioral Phenomenology

Some patients described the behavior as habitual and occurring without clear emotional awareness. Episodes were often reported during low cognitive demand activities, such as when the hands were unoccupied or during monotonous tasks.

Patients with AN frequently referred to a reduced presence of conscious thoughts during the behavior, describing experiences such as “emptiness”, “no thoughts”, or acting “on autopilot”. In several cases, the behavior occurred in response to boredom or the need to focus attention on a sensory stimulus.

## 4. Discussion

### 4.1. Principal Findings

The present study examined the functional meaning of compulsive skin picking in patients with AN within a clinical setting. Across interviews, participants described a relatively consistent sequence: an increase in internal tension, followed by short-term relief after the episode, and subsequently the emergence of self-directed negative emotions.

Skin picking occurred in situations of emotional overload, understimulation, or reduced emotional awareness. It was also frequently connected with eating-related anxiety, dissatisfaction with the body, and, in some cases, hunger-related distress.

These findings point to compulsive skin picking as a behavior that may reflect ongoing emotional and interoceptive dysregulation, rather than representing an incidental comorbid symptom.

### 4.2. Functional Meaning and Negative Reinforcement

The identified emotional sequence is consistent with negative reinforcement models described in the BFRB literature [13,14]. Patients consistently reported escalating internal tension preceding the behavior, temporary relief during or immediately after the episode, and subsequent secondary negative emotions. This cyclical pattern helps explain the persistence of the behavior despite awareness of its negative consequences.

Importantly, within the context of AN, this regulatory mechanism appears embedded in broader emotion regulation difficulties previously described in eating disorders [3,9]. The behavior may therefore represent a repetitive tension-modulating strategy rather than an impulsive or self-injurious act. Unlike intentional self-harm, patients did not describe a primary intention to inflict pain but rather to modulate internal states.

### 4.3. Regulatory and Automatic Components

The analysis revealed the coexistence of two partially overlapping components. First, a regulatory component linked to heightened emotional arousal, and second, a more automatic or habitual component occurring in states of reduced awareness or low stimulation.

For some patients with AN, skin picking followed intense emotional activation and appeared to serve a clear regulatory function. For others, the behavior occurred during boredom, fatigue, or in an autopilot mode, accompanied by reduced conscious thought. Such descriptions resemble phenomena of narrowed awareness or partial dissociation described in the BFRB literature [24,25]. In the context of AN, similar states have been associated with emotion regulation deficits and altered executive functioning [3,9].

These findings suggest a possible progression in which initially tension-reducing behaviors become increasingly automatized over time, appearing even in emotionally neutral contexts. This dual-process pattern aligns with contemporary models of repetitive behaviors integrating affective and habitual mechanisms [11,13].

### 4.4. Body Image and Eating-Related Context

Skin picking episodes were frequently associated with body dissatisfaction, appearance-focused thoughts, and eating-related anxiety. In several AN patients’ narratives, perceived skin imperfections triggered episodes, suggesting that the behavior may be embedded within distorted body image processing rather than representing an isolated impulse-control symptom.

Patients also described episodes in the context of food-related stress, guilt, or tension surrounding meals. This finding indicates that skin picking may be functionally linked to core mechanisms of AN, particularly affect regulation related to body image and eating control [3,4,9]. In this sense, BFRB may represent a behavioral expression of the same regulatory vulnerabilities that maintain restrictive eating patterns.

### 4.5. Metabolic and Interoceptive Context

The findings may also be interpreted within a broader metabolic and interoceptive framework. AN is characterized by chronic energy restriction and altered interoceptive processing, including disturbances in hunger perception and autonomic regulation [5,6]. Under such conditions, internal tension may reflect not only psychological distress but also persistent homeostatic imbalance.

When biological hunger signals are cognitively suppressed due to rigid dietary control, a psychobiological conflict may arise between homeostatic needs and overcontrolled behavior. Patients frequently described bodily activation, tension, and discomfort preceding skin picking episodes. Although the present study did not directly assess metabolic parameters, these experiential accounts are consistent with altered interoceptive awareness documented in AN [5,6].

From this perspective, compulsive skin picking may function as a micro-regulatory strategy aimed at modulating autonomic arousal that cannot be resolved through adaptive nutritional responses. The temporary relief reported by patients aligns with negative reinforcement mechanisms [13,14] but may additionally reflect attempts to regulate internally generated physiological activation associated with energy deficit and renutrition-related stress.

Importantly, this interpretation does not imply direct causality but rather suggests that metabolic and interoceptive dysregulation may increase vulnerability to alternative tension-modulating behaviors. Further research integrating physiological and qualitative methodologies is warranted.

The coexistence of emotional, cognitive, and interoceptive antecedents suggests that compulsive skin picking in anorexia nervosa may operate at the intersection of affective dysregulation and metabolic stress.

The present study did not include a detailed dietary assessment or a quantitative analysis of nutrient intake. Therefore, the potential contribution of specific macro- and micronutrient deficiencies to the observed phenomena remains an important area for future research.

From a nutritional perspective, chronic deficiencies in essential nutrients (e.g., fatty acids, vitamin A, and micronutrients involved in tissue repair) may further compromise skin integrity in individuals with AN, as previously described in studies on dermatological manifestations of eating disorders [15]. Such deficiencies may increase susceptibility to skin damage and delayed healing, potentially reinforcing the cycle of skin picking. This interpretation is also consistent with broader evidence linking nutritional factors with skin condition and repair processes [16]. Although the present study did not directly assess nutritional status, this aspect represents an important biological context for interpreting the observed behaviors.

Severe energy restriction and associated deficiencies in macro- and micronutrients may also affect central nervous system functioning in individuals with AN. Previous research indicates that malnutrition may alter processes related to impulse control, emotional regulation, stress responsiveness, and interoceptive awareness [26].

These neurobiological alterations may increase vulnerability to repetitive and compulsive behaviors, particularly in situations of heightened internal tension. In this context, compulsive skin picking may emerge as a behavior that reflects both altered central regulation and increased peripheral vulnerability of the skin, as described in dermatological manifestations of eating disorders [15].

Thus, the observed behavior may be understood as a multidimensional phenomenon, resulting from the interaction between nutritional deficits, neurobiological dysregulation, and impaired skin integrity.

### 4.6. Clinical Implications for Multidisciplinary Care

The findings have practical implications, especially for multidisciplinary treatment. These implications extend beyond general clinical awareness and may inform specific assessment and decision-making processes within multidisciplinary teams. In psychotherapy, skin picking episodes may signal moments of reduced emotional tolerance and provide entry points for interventions targeting adaptive regulation strategies [3,9]. For physicians, visible skin damage may indicate underlying emotional destabilization rather than solely behavioral dyscontrol.

In nursing practice, the emergence or intensification of episodes may provide early warning signs of emotional dysregulation. From a nutritional perspective, episodes occurring in relation to hunger, meals, or renutrition may reflect regulatory overload associated with metabolic stabilization processes. Routine assessment of body-focused repetitive behaviors may also support ongoing monitoring of treatment-relevant changes during hospitalization.

Some patients reported a subjective reduction in the frequency or intensity of episodes during hospitalization and nutritional stabilization. Although the cross-sectional design does not allow causal inference, this observation may suggest a dynamic relationship between metabolic state and emotional regulation. Incorporating BFRB into routine clinical assessment may support more comprehensive monitoring of treatment progress.

From a practical standpoint, routine assessment of compulsive skin picking may serve as a clinically useful indicator of ongoing dysregulation that is not always captured through standard self-report measures. In particular, the presence, frequency, and contextual triggers of BFRB episodes may provide additional information about moments of reduced emotional tolerance, increased interoceptive distress, or difficulties related to nutritional rehabilitation.

In multidisciplinary treatment planning, these observations may have several implications. For psychotherapists, identifying patterns of skin picking may support the implementation of targeted interventions focused on emotion regulation and distress tolerance. For physicians, visible skin damage may function as an observable marker of internal destabilization, potentially indicating the need for closer monitoring of the patient’s overall clinical status. For dietitians, episodes occurring in proximity to meals or hunger-related states may signal increased regulatory burden associated with nutritional normalization, suggesting the need for adjustments in meal structure or pacing. In nursing care, changes in the frequency or intensity of episodes may serve as early warning signs of emotional deterioration requiring prompt intervention.

Taken together, incorporating BFRB assessment into routine clinical evaluation may enhance interdisciplinary communication and support more individualized and responsive treatment planning.

From a clinical perspective, the present findings may inform the development of more targeted intervention strategies for individuals with anorexia nervosa presenting with compulsive skin picking. Interventions focusing on emotion regulation (e.g., cognitive-behavioral approaches and techniques derived from dialectical behavior therapy) may be particularly relevant, as the behavior appears closely linked to internal tension and affective dysregulation.

Additionally, enhancing interoceptive awareness and the ability to identify and tolerate internal states may reduce reliance on maladaptive regulatory behaviors. Behavioral interventions commonly used in the treatment of body-focused repetitive behaviors, such as habit reversal training and stimulus control techniques, may also be beneficial.

Importantly, addressing underlying nutritional deficits and supporting metabolic stabilization may indirectly contribute to reducing vulnerability to tension-driven behaviors. These approaches may be most effective when implemented within a multidisciplinary treatment framework integrating psychiatric, psychological, and nutritional care.

### 4.7. Limitations and Strengths

Several limitations should be acknowledged. The analyzed subgroup consisted exclusively of female patients, which limits the transferability of the findings to male populations or individuals with more diverse gender identities. Given potential gender-related differences in emotional processing, body image, and the expression of body-focused repetitive behaviors, the present results should be interpreted with caution beyond female clinical samples. Data were based on detailed field notes rather than audio recordings, which may have limited verbatim precision. Although the immediate expansion of notes and independent analysis by two researchers reduced interpretative bias, subtle linguistic nuances may not have been fully captured.

The sample consisted primarily of hospitalized patients from a single clinical center, limiting generalizability to outpatient populations or individuals with milder forms of anorexia nervosa. The study did not include standardized quantitative measures of BFRB severity, interoceptive awareness, or physiological and metabolic parameters. As a result, the proposed links between skin picking behavior, interoceptive dysregulation, and energy deficit remain interpretative and cannot be empirically verified within the present design. The cross-sectional design precludes conclusions regarding directionality between metabolic state and repetitive behaviors.

Nevertheless, the study provides an in-depth functional analysis of compulsive skin picking within the specific clinical context of AN and integrates emotional, cognitive, and interoceptive dimensions. It contributes novel insights into the potential role of BFRB as observable markers of regulatory instability in multidisciplinary care settings.

## 5. Conclusions

Compulsive skin picking in patients with AN may constitute an observable behavioral marker of emotional and interoceptive dysregulation rather than merely a coincidental comorbid phenomenon. The identified pattern, characterized by heightened internal tension followed by transient relief, is consistent with negative reinforcement mechanisms and supports the interpretation of a regulatory function of the behavior.

Within the broader clinical context of AN, which is characterized by chronic energy restriction and altered interoceptive processing, such behaviors may reflect attempts to modulate internally generated physiological and affective arousal. Although causal inferences cannot be established based on the present qualitative design, the findings are consistent with the hypothesis that persistent homeostatic tension associated with restrictive eating patterns may increase vulnerability to alternative tension-regulation strategies.

Routine assessment of body-focused repetitive behaviors in patients with AN may enhance clinical evaluation of ongoing emotional and interoceptive instability, particularly in cases where self-reported distress appears attenuated. Systematic monitoring of these behaviors may facilitate a more comprehensive multidisciplinary approach to care, integrating psychological, nutritional, and medical perspectives.

Future research integrating qualitative methodologies with physiological and metabolic assessments is warranted to further elucidate these mechanisms.

## Figures and Tables

**Table 1:** Sociodemographic and clinical characteristics of participants with anorexia nervosa (N = 33).

Sociodemographic or Clinical Characteristics		N (%)
Eating Disorder Diagnosis	Anorexia nervosa without comorbid eating disorders	26 (78.8%)
	Anorexia nervosa with comorbid eating disorders *	7 (21.2%)
Sex	Female	33 (100%)
Age	16–18 years	5 (15.2%)
	18–24 years	18 (54.5%)
	25–34 years	10 (30.3%)
Educational Level	Primary education	5 (15.2%)
	Secondary education	9 (27.3%)
	Higher education	19 (57.5%)
Place of Residence	Rural area	5 (15.2%)
	City < 50,000 inhabitants	3 (9.1%)
	City 50,000–200,000 inhabitants	6 (18.2%)
	City > 200,000 inhabitants	19 (57.5%)

* Anorexia nervosa with bulimia nervosa (n = 4); anorexia nervosa with binge-eating episodes (n = 2); anorexia nervosa with orthorexia (n = 1).

**Table 2:** Dominant emotional transition patterns associated with compulsive skin picking.

Antecedent Emotional State	Immediate Change	Example Quotation
Stress/tension	Relief/calming	“Before, I feel this growing tension and restlessness inside me, like something is building up and I cannot calm down. When I start picking, it gives me a sense of relief and my body feels calmer, but it only lasts for a short time.”
Anxiety	Relief/calm/neutrality	“I feel anxious and restless before it starts, like I cannot relax or focus on anything else. When I begin picking, it helps me calm down and for a moment I feel more neutral, like the tension disappears.”
Anger/frustration	Self-directed anger/Reduction in arousal	“I feel anger and frustration, especially when I notice imperfections on my skin. When I start picking, the tension goes down, but afterwards I feel angry at myself for doing it again and not being able to stop.”
Sadness/uncertainty	Persistent sadness or guilt/Partial relief	“I feel sadness and sometimes uncertainty before I start, like I don’t really know what I’m feeling but it’s uncomfortable. Picking gives me a bit of relief, but afterwards the sadness comes back together with guilt.”
Boredom	Soothing/satisfaction	“When I feel bored or empty, I start picking without thinking much about it. It gives me something to focus on and feels a bit satisfying, like it fills the emptiness for a moment.”
No clear emotion	Disappointment	“Sometimes it just happens automatically and I don’t notice when it starts. Only afterwards I realize what I’ve done, and then I feel disappointed and frustrated with myself.”
Sensory fascination	Distress about effects/Engagement	“I feel drawn to small imperfections, like I need to touch or fix them. While I’m doing it, I feel focused on the sensation, but afterwards I get irritated and nervous when I see the damage to my skin.”

**Table 3:** Emotional states preceding skin picking episodes (n = 33).

Emotional State	Participants n (%)	Example Quotation
Stress/tension	19 (57.6%)	“Before—stress and agitation.”
Anxiety/inner tension	8 (24.2%)	“Anxiety and restlessness before it starts.”
Anger/frustration	7 (21.2%)	“I get angry when I see imperfections.”
Sadness/guilt	5 (15.2%)	“I feel bad and then I start picking.”
Boredom/emptiness	4 (12.1%)	“When nothing is happening, I do it.”
Automatic/unclear emotion	6 (18.2%)	“Nothing specific it just happens.”
Sensory interest	2 (6.1%)	“I want to peel the scab.”

**Table 4:** Situational contexts associated with skin picking episodes (n = 33).

Context	Participants n (%)	Example Quotation
Emotional stress/overload	19 (57.6%)	“After a stressful day, I start picking.”
Evening/before sleep	10 (30.3%)	“It usually happens when I lie in bed.”
Monotonous activity/free hands	8 (24.2%)	“When I watch something and my hands are free.”
Private setting	11 (33.3%)	“Only when I’m alone in my room.”
Rumination/loss of control	7 (21.2%)	“When I keep thinking and can’t stop.”
No clear pattern	6 (18.2%)	“It happens randomly.”

**Table 5:** Physiological sensations associated with skin picking episodes (n = 33).

Physiological Sensation	Participants n (%)	Example Quotation
No noticeable bodily sensation	15 (45.5%)	“Nothing specific, I just start doing it.”
Cardiovascular arousal	10 (30.3%)	“My heart starts beating faster.”
Muscle tension	7 (21.2%)	“I feel tension in my muscles.”
Autonomic symptoms (heat/sweating)	8 (24.2%)	“I feel warmth and sweating.”
Complex arousal symptoms	4 (12.1%)	“Dizziness and difficulty breathing.”

**Table 6:** Perceived emotional effects of skin picking (n = 33).

Perceived Effect	Participants n (%)	Example Quotation
Temporary relief	24 (72.7%)	“It calms me down for a moment.”
No change	5 (15.2%)	“It doesn’t help.”
Ambivalent effect	2 (6.1%)	“It helps, but only briefly.”
Increased discomfort	2 (6.1%)	“I feel worse afterwards.”

**Table 7:** Emotional experiences following skin picking episodes.

Emotional Response	Example Quotation
Relief/calming	“Afterwards, I feel calmer.”
Guilt/shame	“I regret doing it.”
Self-directed anger	“I’m angry at myself again.”
Sadness	“I feel bad and disappointed.”
Neutrality	“I don’t feel anything.”
Irritation/concern about damage	“I’m upset when I see the wound.”

**Table 8:** Cognitive experiences reported during skin picking episodes (n = 33).

Category	Participants n (%)	Representative Quotations
No thoughts/automaticity (n = 14)	14 (42.4%)	“I didn’t think about anything.” “Nothing.” “I had no thoughts.” “I don’t remember.” “I just started doing it.” “I had thoughts, but automatically.”
Urge-related thoughts focused on the body (n = 7)	7 (21.2%)	“It’s just tension and I have to act.” “I have to squeeze it.” “I need to get rid of the skin.”
Escape and tension-reduction thoughts (n = 6)	6 (18.2%)	“I want to detach.” “I don’t know what to do; I must control it somehow.” “I need relief.” “It was something like meditation—I wasn’t thinking.”
Appearance-focused thoughts (n = 3)	3 (9.1%)	“This is how it looks and it should stop.” “I am worthless until I fix it.”
Self-critical thoughts (n = 5)	5 (15.2%)	“I am hopeless and weak.” “I wish I could finally stop.” “I know I shouldn’t be doing this.”
Overthinking/cognitive overload (n = 3)	3 (9.1%)	“It’s hard to distinguish, but it was a flood of thoughts.” “I kept analyzing different situations.”
Rationalizing thoughts (n = 2)	2 (6.1%)	“If I do it, I’ll feel better.” “If I press it, the feeling will disappear.”

## Data Availability

The data supporting the findings of this study are not publicly available due to privacy and ethical restrictions related to the clinical nature of the data. De-identified data may be available from the corresponding author upon reasonable request and with permission of the institutional ethics committee.

## Abbreviations

The following abbreviations are used in this manuscript:

AN	Anorexia Nervosa
BFRB	Body-Focused Repetitive Behaviors
HPA axis	Hypothalamic–Pituitary–Adrenal Axis
